# Enhanced Medical and Community Face Masks with Antimicrobial Properties: A Systematic Review

**DOI:** 10.3390/jcm10184066

**Published:** 2021-09-09

**Authors:** Katy Stokes, Roberto Peltrini, Umberto Bracale, Marcella Trombetta, Leandro Pecchia, Francesco Basoli

**Affiliations:** 1 School of Engineering, University of Warwick, Coventry CV4 7AL, UK; Katy.Stokes@warwick.ac.uk; 2 Department of Public Health, Federico II University Hospital, 80131 Naples, Italy; roberto.peltrini@gmail.com; 3 Department of Advanced Biomedical Sciences, Federico II University Hospital, 80131 Naples, Italy; umbertobracale@gmail.com; 4 Department of Engineering, University Campus Bio-Medico di Roma, 00128 Rome, Italy; m.trombetta@unicampus.it

**Keywords:** antimicrobial, personal protective equipment, face masks, filtering facepiece respirator, COVID-19, pandemic

## Abstract

Face masks help to limit transmission of infectious diseases entering through the nose and mouth. Beyond reprocessing and decontamination, antimicrobial treatments could extend the lifetime of face masks whilst also further reducing the chance of disease transmission. Here, we review the efficacy of treatments pertaining antimicrobial properties to medical face masks, filtering facepiece respirators and non-medical face masks. Searching databases identified 2113 studies after de-duplication. A total of 17 relevant studies were included in the qualitative synthesis. Risk of bias was found to be moderate or low in all cases. Sixteen articles demonstrated success in avoiding proliferation (if not elimination) of viruses and/or bacteria. In terms of methodology, no two articles employed identical approaches to efficacy testing. Our findings highlight that antimicrobial treatment is a promising route to extending the life and improving the safety of face masks. In order to reach significant achievements, shared and precise methodology and reporting is needed.

## 1. Introduction

The present pandemic due to coronavirus disease (COVID-19) significantly impacted the health of millions of people and highlighted weaknesses in the personal protective equipment (PPE) supply chain around the world. Face masks have been used in medical settings for infection prevention for decades, being one of the most important countermeasures in mitigating high risk of droplet and aerosol transmission of pathogens in health care settings [1]. Medical masks and FFP2 or N95 respirators are recommended for Health Workers (HWs) when providing care to suspected or confirmed COVID-19 patients, especially if performing aerosol generating procedures, which requires respirators to be worn continuously [2]. Patients must wear medical face masks for in person care to control sources of infection [3]. Expanded use of masks has resulted in increased wear time and use without training. Although there is precedent for mask wearing among the general public in Asian countries such as China, South Korea and Japan, this only became a global strategy in reaction to the spread of severe acute respiratory syndrome coronavirus 2 (SARS-CoV-2). Mask wearing is generally considered a low risk and low-cost approach for disease control among the general public, facilitating source control from asymptomatic persons unknowingly transmitting virus. It is accepted that non-medical (community) masks are generally sufficient for this purpose, with members of the public having a lower transmission risk relative to that of HWs. However, certain designs of community masks are considered unfit for use (e.g., those containing respiratory exhalation valves do not offer source control) and use of medical grade PPE by the general population contributed to shortages [4].

In this context, many researchers are investigating the reprocessing and reuse of masks and filtering facepiece respirators (FFPs/FFRs), focusing on meeting regulatory standards and using readily available equipment present in hospitals. Decontamination is of paramount importance to any PPE re-use. Research in this area grew so fast in reaction to COVID-19 that literature reviews became available within one year [5,6,7,8]. Reprocessing or decontamination must ensure devices keep their original properties (e.g., filtering and breathability), functional integrity, shape and there must be no residual toxicity for the wearer [9,10]. Antimicrobial enhanced fabrics could be used to engineer masks and respirators allowing not only a longer lifespan of the mask, but also the possibility to exploit novel routes for mask decontamination. Antimicrobial systems can be broadly grouped into categories, as reported in a recent revision of the literature on PPE for health applications: [11] metal oxides and nanoparticles; salt compounds; graphene-based materials; quaternary ammonium compounds (QACs); N-halamine-based compounds; and naturally derived antimicrobial agents. Of these, nanoparticles, such as copper oxide, graphene oxide nanosheets [12] and plant extracts [13] have been investigated for decontamination methods.

There is no comprehensive systematic review available for masks fabricated with antimicrobial properties, their efficacy against bacteria or viruses or the possibility of reprocessing with the necessary durability and safety. Current standard methods for testing efficacy of antimicrobials are typically specific to the pathogen, i.e., bacterial (ISO 20743, AATCC TM100), viral (ISO 18184) or fungal (ISO 13629). General chemical safety assessments such as REACH chemical safety have limited application to modified fabrics for masks. Any recommendations concerning the development and production of PPE requires access to the best available evidence. Therefore, the present work provides a systematic review of the literature addressing antimicrobial materials and treatments for medical face masks, FFRs and community masks. The aim is to provide evidence-based recommendations on antimicrobial treatments for respirators and masks, especially regarding efficacy, reliability and safety to the wearer, and their possible role in facing the current pandemic and future healthcare crises.

## 2. Methods

### 2.1. Search Strategy and Selection Criteria

This study was conducted in accordance with the preferred reporting items for systematic reviews and meta-analyses (PRISMA) guidelines [14]. The protocol was designed prospectively and is available upon request. Studies were included if they met the following criteria: original peer-reviewed studies describing “augmented” masks by antimicrobial agents reporting at least one laboratory test in order to assess the efficacy.

MEDLINE and Embase databases were searched to identify studies from 1 January 2010 till 1 January 2021. Bibliographies of relevant articles were assessed as a secondary source of studies. The literature search was performed and verified by two independent reviewers using the index terms grouped in three categories: device (respirator OR mask OR filtering OR nonwoven OR fabric OR electro AND spun OR textile OR personal AND protection AND equipment), type of active augmentation (antimicrobial OR antiviral OR nanoparticles OR nanotechnology OR viricidal OR biocidal OR bactericidal OR inactivation) and organism affected (COVID-19 OR bioaerosol OR airborne OR coronavirus OR virus OR respiratory AND infections).

Two authors independently evaluated all retrieved studies against the eligibility criteria and divergent opinions were resolved, achieving consensus through discussion with a third author. Articles were excluded if there was not sufficient documentation. Reviews, duplicate publications and editorials were also excluded.

### 2.2. Data Analysis

Data were extracted independently by two reviewers and entered into standardized spreadsheets. Any disagreement was resolved, achieving consensus via discussion with a third reviewer. The following data were extracted: type of article, publication year, type of substrate, type of antimicrobial system, integration methodology, viruses and/or bacteria tested and methods, antimicrobial efficacy results and secondary outcomes.

### 2.3. Outcomes

The main outcome was antimicrobial efficacy of antimicrobial treatments applied to PPE, measured by Logarithmic Reduction Value (LRV) of Colony Forming Units (CFU). This review also focused on methodological aspects, such as methods employed to assess antimicrobial efficacy, antimicrobial technology employed, how the treatment was integrated with the production process, which comparator was employed in each study, which pathogen was used and how it was applied to the PPE. Finally, a number of secondary outcomes were systematically investigated, aiming at assessing the impact of antimicrobial treatment on PPE fundamental properties, including: breathability; filtering capacity; reusability; impact on PPE cost/production; stability and durability of treatment; and safety for the wearer (e.g., toxicity via inhalation of antimicrobial treatment substances, skin irritations or respiratory inflammation).

### 2.4. Risk of Bias

Due to lack of standardized tools for assessing study results and risk of bias in this field, we employed previously developed objective assessment criteria [10,15]. This consisted of a predetermined evaluation matrix, containing information on study design, methodological consistency, population heterogeneity, sampling bias and selective reporting (Table S3 in Supplementary Materials). We adapted the original matrix, removing categories not relevant to this review. The assessment was made by two reviewers independently and divergences were overcome with consensus.

### 2.5. Role of Funding Source

The funder was involved in defining the scope of the work. Study design, data collection, data interpretation and report writing were completed independently of the funder.

## 3. Results

**Figure 1:**
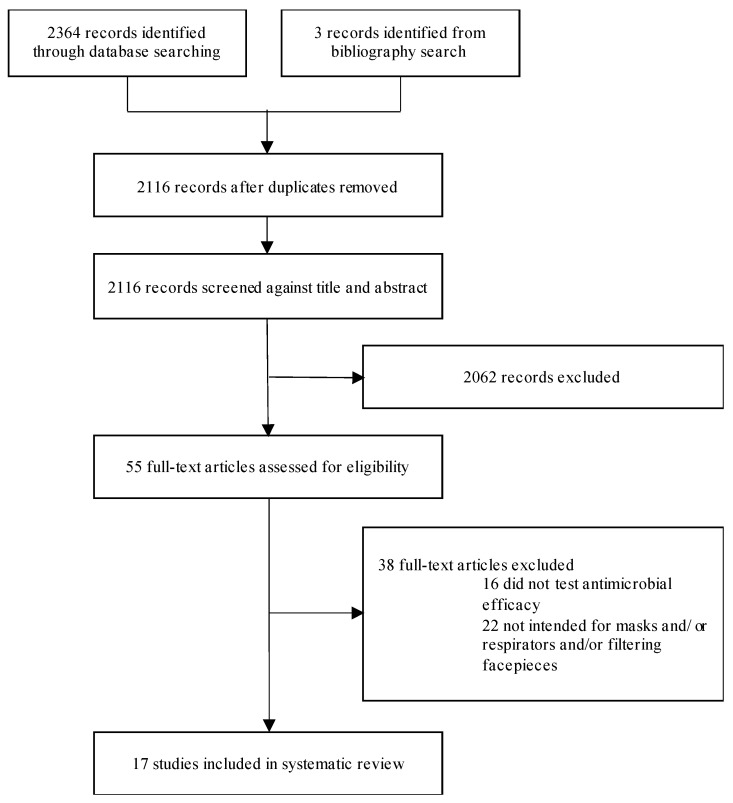
Study selection. PRISMA flow diagram of search and screening process.

Searching MEDLINE and Embase yielded 2364 titles. After duplicate removal, 2116 titles/abstracts were screened, with 1982 excluded. Among the remaining 55 full-texts screened for compliance with the eligibility criteria, only 17 studies met the eligibility criteria and reported sufficient experimental results and methodological details for inclusion in the final analysis. Excluded full texts and reasons for exclusion are provided in Table S1. A flow chart representing the screening process is given in Figure 1. Table 1 contains a summary of the characteristics of the included studies covering three main areas: types of antimicrobial systems used, antimicrobial efficacy testing and results. A detailed account of all extracted information is provided in Table S2. Risk of bias was moderate or low in all studies, covering the following areas: study design, methodological consistency, population heterogeneity, sampling bias and selective reporting (Tables S3 and S4).

One study investigated commercially available antimicrobial FFRs [16], all other studies proposed novel antimicrobial systems [17,18,19,20,21,22,23,24,25,26,27,28,29,30,31,32]. A total of 15 studies concerned antimicrobial properties pertained through modification of pre-existing masks, filters or fabric substrates [16,17,18,19,20,21,22,24,25,26,27,29,30,31,32]. Two studies introduced antimicrobial compounds directly during fibre synthesis [23,28].

In 16 studies laboratory pathogen strains were used to investigate antimicrobial efficacy using methods of: inoculation/incubation with the test system [17,18,21,24], bioaerosol challenge [16,19,20,25,27,30,31] or both [22,23,26,28,32]. Only one clinical study, where bacteria were recovered from masks worn by volunteers, compared bacterial growth between worn/unworn masks [29]. Many studies compared antimicrobial activity towards Gram-negative and Gram-positive bacteria, most commonly investigated bacteria were by far Gram-negative *Escherichia coli (E. coli)* and Gram-positive *Staphylococcus aureus (S. aureus*) [18,20,21,22,23,27,28]. In order to measure activity against viruses, four studies used surrogates: three investigated the MS2 bacteriophage (MS2) [16,25,31] and one utilised extracellular vesicles as ‘virus like particles’ [26]. Five studies analysed viruses directly, employing a variety of influenza strains [17,30,32]. Only two studies investigated both bacteria and viruses [25,26]. No articles evaluated SARS-CoV-2, though viruses with comparable characteristics were considered.

**Figure 2:**
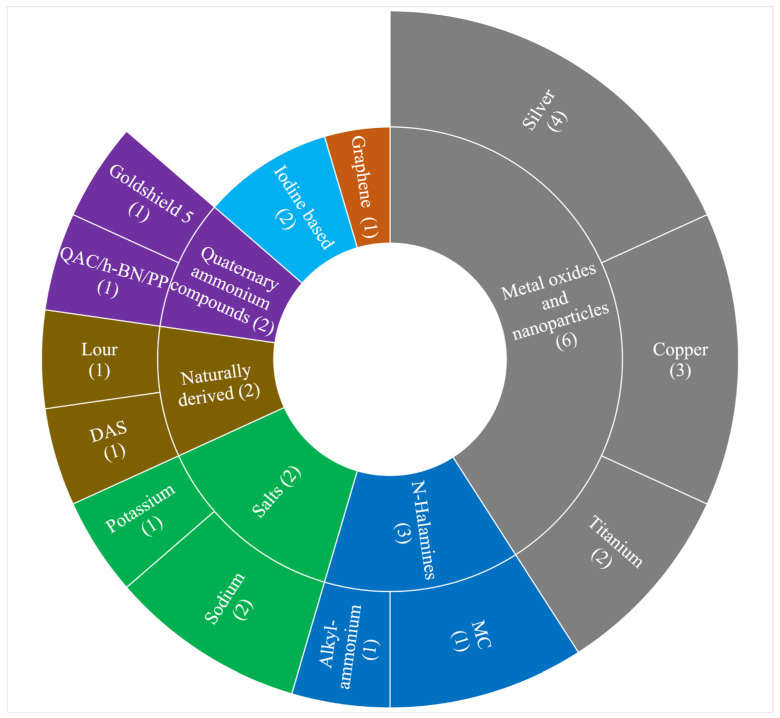
Classes of antimicrobial system.

A breakdown of the antimicrobial systems is given in Figure 2. Six studies utilized metal oxides and nanoparticles [16,17,18,20,24,25,26]. Borkow et al. [17] reported a copper oxide system applied to layers of an N95 FFR. A statistically significant contact inactivation of aerosolized human influenza A virus (H1N1) (2.88 log) and avian influenza virus (H9N2) (3.13 log) was granted relative to an untreated control, using a modified ASTM Method F 210101. Kumar et al. [26] coated polypropylene nonwoven fabrics of surgical masks with a composite of Shellac and copper nanoparticles. Their photocatalytic mechanism granted increased surface hydrophobicity and rapid temperature increase under solar illumination, achieving a four-log reduction of CFUs of *E. coli* relative to untreated fabric. A third study compared a NIOSH-approved N95 FFR with three manufacturer supplied prototype antimicrobial masks utilizing: silver-copper, EnvizO3-Shield technology (reactive oxygen species), TiO_2_ and iodine-activated resin [16]. Following bioaerosol challenge with droplet nuclei containing MS2, three fabrics (TiO_2_ which could not be tested in those conditions) showed a higher log_10_ reduction than the N95, but only under conditions of 37 °C 80% RH. Further, the reduction was only significant for the iodinated system (3.7 log reduction of MS2). Lore et al. [25] also investigated an iodine-based antimicrobial treatment for respirators. However, no viability reduction was observed in this case, when challenged with three bioaerosols; MS2 bacteriophage virus, *Bacillus atrophaeus* vegetative bacteria and endospores. Other metal compounds included silver nitrate and titanium dioxide, which were found to grant a 100% reduction in viable *E. coli* and *S. aureus* recovered from the modified FFR, compared with an increase in viable bacteria from the untreated control FFR [18]. A silver based antimicrobial system was also investigated by Hiragond et al.; a commercially available mask augmented with colloidal silver [20]. Again, significant antibacterial activity (relative to untreated control) was seen against *E. coli* and *S. aureus* using a well-diffusion assay. A third study coated silver nanoparticles onto the filtering layer of a N95 FFR [24]. Bacterial growth (*Pseudomonas aeruginosa* and *S. aureus*) was strongly inhibited on coated fabric. Moreover, Field Emission Scanning Electron Microscopy (FE-SEM) showed minimal attachment of cells to treated fabric, no growth of bacteria colonies and cell debris, indicating cellular disruption due to the antimicrobial coating.

Internal segments denote the broad class of antimicrobial system and the corresponding number of studies, external segments display the specific compounds and the number of times they were employed across the study pool. Most antimicrobial systems were evaluated by only one study.

Antimicrobial properties of salt compounds were evaluated by two studies [27,30], in both studies transmission electron microscopy (TEM) highlighted structural damage and morphological changes in test pathogens, attributed to contact with the natural salt recrystallization process. Quan et al. applied a sodium chloride (NaCl) salt coating to a surgical mask polypropylene filtering layer [30]. Aerosolized viral strains: H1N1, PR/34 H1N1 and VN/04 H5N1 applied to coated fabric were determined to be inactive by TEM, due to hyperosmotic stress upon the viral envelope. A second study also evaluated NaCl, alongside potassium sulphate (K_2_SO_4_) and potassium chloride (KCl) [27]. Bacteria strains: *Klebsiella pneumoniae*, methicillin-resistant *S. aureus*, *E. coli*, *Pseudomonas aeruginosa* and *Streptococcus pyogenes* showed time-dependent inactivation for all salt coatings. Best performance was seen with a three-layer NaCl filter, granting a 4log reduction within 30 min of aerosol exposure. Bacterial inactivation was confirmed in vivo using a mouse infection model.

In one study, laser-induced graphene (LIG) was synthesized into fabric using Polyimide substrate [28]. The antibacterial properties were tested alongside commercial samples of activated carbon face masks (activated carbon fibre (ACF)) and surgical masks (melt-blown fabric, MBF), by submerging in *E. coli* suspension. A CFU assay showed 0.73 log reduction of *E. coli* after 8 h. The antibacterial activity of LIG was found to be high, 81.57%, compared to ACF (2.00%), and MBF (9.13%). Hydrophobic and hydrophilic LIG were compared and exhibited similar antibacterial activity. The authors propose this happens through different mechanisms; the former due to the abundant oxygen-containing functional groups such as −COOH and −OH, that may cause loss of intracellular substances due to charge transfer, the latter due to dehydration.

Two QACs were evaluated [19,21]. Tseng et al. [19] applied Goldshield 5 (QAC based commercial detergent), to a surgical face mask. Over 99.3% antibacterial efficiency was seen when aerosolized bacteria (*Acinetobacter baumannii*, *Enterococcus faecalis* and *S. aureus*) challenged the mask surface. Xiong et al. [21] modified the PP layer of a surgical mask using QAC//Hexagonal Boron Nitride/PP (QAC/h-BN/PP), forming a nanocomposite, activated surface. *E. coli* and *S. aureus* were incubated with test fabric samples following standard methods (ISO 22196 and JIS Z 2801). Antimicrobial rates of the QAC/h-BN/PP samples were 99.3% (*E. coli*) and 96.1% (*S. aureus*), based on optical density of recovered bacteria. A so-called ‘contact killing’ mechanism was confirmed by zone-of-inhibition testing, i.e., the system did not release biocidal compounds.

N-halamine based biocidal systems were employed in three studies [22,23,32]. Majchrzycka et al. [23] used two inorganic carriers (bioperlite and biobentonite) to functionalize filtering nonwovens with an alkylammonium biocide (during fibre formation). Time dependent CFU assays showed biobentonite had low bacteriostatic and bactericidal activity towards *E. coli* and *S. aureus*. In contrast, bioperlite granted high biostatic and biocidal effects. Best performance was seen with 15/20% bioperlite, after six hours of incubation (100% reduction of *S. aureus*). Two studies investigated the N-halamine compound 1-Chloro-2,2,5,5-tetramethyl-4-imidazolidinone (MC) [22,32]. MC was coated onto melt-blown nonwoven fabrics used for surgical face masks [22] and N95 respirators [22,32]. Both studies employed two testing methods: sandwich test and bioaerosol challenge (ASTM Method F 2101.01). Demir et al. [22] evaluated bacterial LRV following sandwich testing, finding 6.1log for *E. coli* (10 min) and 6.26log for *S. aureus* (5 min). All bacteria collected from fabrics following aerosol exposure were inviable. Ren et al. [32] investigated MC’s antiviral properties, sandwich testing showed complete inactivation of H1N1 virus (10 min). Even 0.1% *w/v* MC samples showed significant viral reduction. Against aerosolized H1N1, MC samples caused complete virus inactivation.

Two studies investigated naturally derived antimicrobial agents [29,31]. Duong-Quy et al. [29] reported a novel face mask (Lamdong Medical College (LMC) mask) containing an antimicrobial agent derived from the leaf oil of *Folium Plectranthii amboinicii* (Lour), a traditional Vietnamese medicinal plant used to treat upper respiratory infections, bronchitis and gastrointestinal infections. The LMC mask and a four-layer activated carbon surgical mask (positive control) were worn by randomized volunteers, followed by laboratory analysis of recovered bacterial growth. Antibacterial activity of the LMC mask was not statistically different to the positive control. Woo et al. [31] investigated dialdehyde starch (DAS), seeking alternatives to aldehyde antimicrobials, which are highly toxic to humans. Commercial filters, such as two cellulose filters (CFs) commonly used for air cleaning and a polypropylene FFR (PF), were modified with DAS aqueous suspension at different concentrations. The antimicrobial assessment was made using a nebulized solution of MS2 bacteriophage and artificial saliva, to emulate aerosols produced from sneezing or coughing. Relative survivability (RS) of MS2 viruses on filters treated with different concentrations of DAS suspension showed a clear biocidal effect for all the filters, with RS decreasing with increasing concentration of DAS.

**Figure 3:**
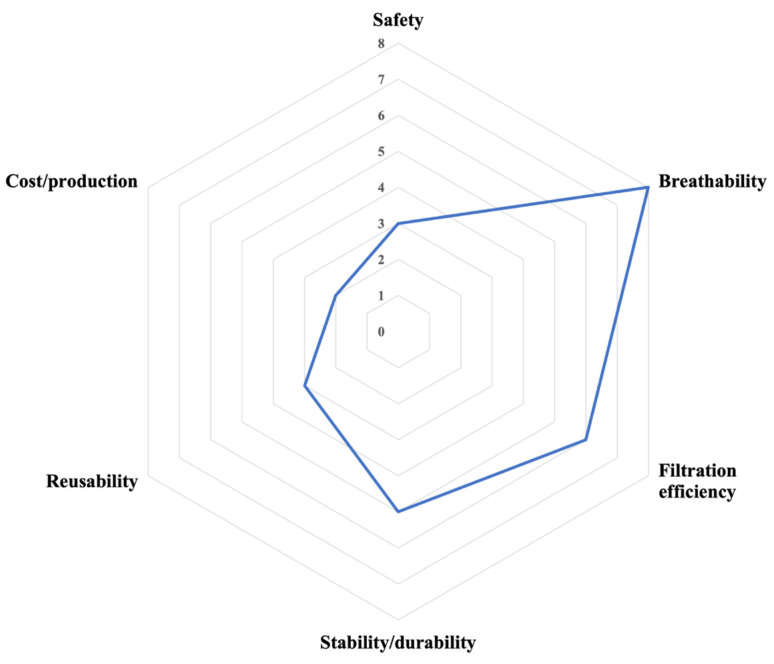
Number of studies considering other PPE properties beyond antimicrobial efficacy.

Table 2 contains a summary of the secondary outcomes addressed in the study pool. Three studies did not evaluate secondary outcomes [20,24,32]. The frequency of secondary outcomes investigated is given in Figure 3. No significant difference [22,27,28] or very slight increase [26] in pressure drop across treated vs. untreated substrates was seen, taken to suggest acceptable levels of breathability. Woo et al. [31] compare similarly treated CF and PF substrates. CFs showed improvement in both pressure drop (lowered) and filtration efficiency (increased) when treated with DAS, with no improvement seen for PFs. A single study reported reduced filtration efficiency after coating of commercially available NIOSH FFRs (N95 and P95) [25]. Two studies found no change in filtration efficiency following antimicrobial treatment [23,26], whereas salt coating increased filtration efficiency in one report [27]. Three studies evaluated toxicity [17,18,29]. One study evaluated particles released from the antimicrobial system, the authors demonstrated that copper eluted from their test mask was within the permissible exposure limit [17]. In contrast, Li et al. asked 20 volunteers to wear their nanoparticle-treated facemask, finding no reports of inflammation or itching after wearing. One study utilised a biomarker for respiratory inflammation to detect any inflammation potentially caused by wearing [29]. No formal cost analysis was offered, but several studies made statements about advantageous low-cost or easy production [17,20]. Durability/stability in terms of shelf life or storage was evaluated in four studies [19,26,27,30]. Tseng et al. found that their GS5 ‘decontamination effect’ lasted a week after initial coating, concluding this would reduce cleaning costs and increase feasibility [19]. Kumar et al. claimed ‘self-cleaning’ properties (via nonwetting surface properties) reduced risk of exposure to pathogens on disposal [26]. Three studies investigated stability of the antimicrobial system under varied environmental conditions, to address storage considerations [16,27,30]. All systems were found to be stable to high temperature and humidity, taken to suggest safe long-term storage and reuse.

Fourteen studies considered secondary outcomes of which just three deal with a very important parameter which is safety for the wearer.

## 4. Discussion

The purpose of this literature review was to determine the efficacy of antimicrobial treatments applied to medical or community face masks. We focused on antimicrobial efficacy, methodological procedure and the impact of modifications on essential PPE properties. A total of 17 studies were included; of the excluded texts, 22 did not concern PPE, while 16 did not report antimicrobial efficacy. Overall, antimicrobial treatments were found to be effective. Yet, it is crucial to recognize the huge heterogeneity among studies, including technology employed, integration method, efficacy testing methods, challenge pathogens and control masks/fabrics. This is likely due to the fact that this is the first study systematically reviewing literature focusing on antimicrobial treatments for PPE. It is urgent to achieve standard methodology, in order to regulate community masks claiming to possess antimicrobial properties, becoming increasingly available on the market. Evidently, there is no standard method for assessing antimicrobial properties.

The very limited number of articles relating directly to antimicrobial systems for masks, relative to many focused on more general antimicrobial modified fabric, is noteworthy. We observed many articles investigating antimicrobial properties of augmented materials, postponing considerations of the final application to future research [33]. Other applications included skin wound care [34,35], water purification [36,37], air filters [38,39] and antimicrobial surfaces [40]. Unsurprisingly, most studies were published following outbreaks of epidemic-prone respiratory pathogens such as Avian influenza virus (H5N1) (2003) [41], H1N1 swine flu (2009/10) [42] and SARS-CoV-2 [43,44]. The majority of antimicrobial agents identified carry a body of evidence supporting their mechanism and utility [45]. The antimicrobial activity of copper and silver, especially in nanoparticle form, is well documented, including towards coronaviruses [46,47,48]. Indeed, N-halamines, graphene and QACs have proved effective against a broad spectrum of microorganisms, with long-term stability and durability [49,50,51]. The mechanism of graphene’s antimicrobial action remains controversial, as discussed in Seifi et al.’s review of antibacterial properties of graphene. Several possible mechanisms were identified, including membrane stress, charge transfer, entrapment, oxidative stress, self-killing and photothermal, which may occur alone or in combination [51]. Photoactive chemicals producing reactive oxygen species are also considered effective and durable candidates for fabrication of antimicrobial materials [52,53].

The relative scarcity of research tackling antimicrobial systems for masks is compounded by a lack of shared procedures for evaluating efficacy and safety. Indeed, test methods were highly heterogenous amongst the identified studies, with antimicrobial assays varying from study to study (e.g., Sandwich test, ASTM Method F2101.01, CFU assay, zone of inhibition assay, etc.). This was also reflected in the challenge pathogens, varying greatly among bacteria, viruses and virus surrogates. Although all methods were scientifically valid, heterogeneity prevented direct comparison. In total, 16 of the 17 articles reported high efficacy of their augmented mask systems in either preventing proliferation or directly eliminating viral or bacterial pathogens. Of all systems considered, only one did not prove effective.

Interestingly, no studies attempted complete analysis of the mask system, covering fundamental properties of an airway protection device, i.e., filtering capacity, breathability (permeability to air, pressure drop), toxicity and longevity of the antimicrobial system and cost. Many studies evaluated some of these properties, while others provided antimicrobial efficacy results alone, leaving further analysis entirely to future work. An important aspect, safety of the product for the wearer, was addressed only in three studies, each with unique approaches. Only one study considered the possibility of reprocessing, authors claimed that their photoactive mask was able to ‘self-sterilize’ under solar irradiation, whilst maintaining its antiviral properties. Several articles discussed shelf life and reuse of their mask system, granted through the persistently active antimicrobial system, even when subject to variable humidity and temperature.

To improve homogeneity, considering the availability of community masks claiming antimicrobial efficacy, it is paramount that standards for testing antimicrobial efficacy and safety are created. Such standards should include well-defined results reporting, including appropriate comparators, considering not only the maximum value of LRV of CFU, but also reporting LRV at different time points, i.e., giving indications on the efficacy during wearing. Additionally, the maximum duration of the antimicrobial protection should always be addressed. The antimicrobial efficacy should be considered against viruses and bacteria in aerosolized and inoculated forms. All identified studies focused on reduction of transmission of airborne pathogens, none addressed transmission from contact with fluid secretions, i.e., touching of masks with contaminated hands, a major issue for non-professional users.

Moreover, safety is paramount, to avoid toxicity or adverse effects to the wearer, pre-existing standards must be met (e.g., REACH regulations from EU). Further, impact of modifications on key properties must be understood, namely filtration efficiency (e.g., ISO 21501-4; EN 14683:2019, EN 13274-7:2019; ASTM F2299) and breathing resistance (e.g., EN 14683:2019 (Annex C); ASTM D737; ISO 9237:1995). Lastly, treatment persistence should be considered and quantified (including limiting number of decontamination cycles in the case of reusable systems), including indications of storage/shelf life under normal conditions.

## Figures and Tables

**Table 1:** Characteristics of included studies.

	Substrate	Antimicrobial System	Antimicrobial Efficacy Testing Methods	Pathogen(s) Used in Testing	Comparators/Controls	Antimicrobial Efficacy Results
Borkow et al. (2010) [17]	NIOSH N95 FFP	Copper oxide	Bioaerosol challenge, bacterial Filtration Efficacy	Viral	Untreated	Significant, higher, direct contact inactivation in test masks than control
Li et al. (2006) [18]	FFP	Silver nitrate, titanium dioxide nanoparticles	Inoculation of fabric with pathogens	Bacterial	Sterile FFP	Control mask: Increase in viable bacteria. Test mask: 100% reduction in viable bacteria
Zheng et al. (2016) [24]	NIOSH 3M N95	Silver nanoparticles	Bacterial growth track, FESEM	Bacterial	Untreated	Bacterial growth effectively inhibited. FESEM: Few bacterial cells intact, debris on treated surface
Hiragond et al. (2018) [20]	Surgical face mask	Silver nanoparticles	Well diffusion assay	Bacterial	Untreated	Inhibition zone of treated masks significantly higher than control
Rengasamy et al. (2010) [16]	4 FFRs	Silver-copper, EvixO_3_-Shield, Iodine, Titanium dioxide	Bioaerosol challenge. Conditions: (1) 22 °C 30% RH for 0, 8, 20 h, (2) 37 °C 80% RH for 0, 2, 4 h	Viral	Equivalent FFR	Conditions: (1) no significant difference to control (2) Silver-copper and EvixO_3_-Shield technology higher log_10_ reduction than control. Highest: EvixO_3_-Shield technology
Kumar et al. (2021) [26]	PP nonwoven	Copper nanoparticles	Bacterial inoculation, bacterial live dead assay, bioaerosol challenge	Bacterial, viral	Untreated	4-log reduction in *E. coli* CFUs. Live/dead assay indicates >99.99% reduction of *E. coli*. VLP concentration decreased by 2–3 log
Lore et al. (2012) [25]	4 NIOSH FFRs	Iodine-based	Bioaerosol challenges	Bacterial, viral	Equivalent FFRs	No detectable antimicrobial properties in test masks compared with conventional
Rubino et al. (2020) [27]	Surgical masks	Sodium chloride, potassium sulphate potassium chloride	Bioaerosol challenge, TEM, in vivo mouse model	Bacterial	Untreated	Physical damage to pathogens. Time-dependent bacterial inactivation. Infected mice lost less body weight and had lower concentrations of lung bacteria than those infected from control
Quan et al. (2017) [30]	PP microfiber filter	Salt: Sodium chloride	Bioaerosol filtration efficiency, TEM	Viral	Untreated	All challenge viruses were inactivated. Evidence that this is due to hyperosmotic stress on viral envelope
Huang et al. (2020) [28]	Prototype	Inherent from LIG	Bacterial live/dead assay, SEM, bioaerosol collection	Bacterial	Commercial filter layer	Antibacterial activity against *E. coli*: LIG: 8157%, ACF: 2.00%, MBF: 9.13%. SEM: surface disruption bacteria. Aerosolized bacterial efficiency 88.89%. *E. coli* viability: 0.73 log reduction
Tseng et al. (2006) [19]	Surgical mask	Goldshield 5	Bioaerosol challenge	Bacterial	Untreated	>99.3% antimicrobial efficiency against bacteria on mask surface for all test pathogens
Xiong et al. (2021) [21]	Prototype	QAC with boron nitride nanoparticles	Incubation with bacteria	Bacterial	Untreated	Antibacterial rate 99.3% for *E. coli* and 96.1% for *S. aureus* through ‘contact killing’ mechanism
Majchrzycka et al. (2012) [23]	PP nonwoven	Alkylammonium	Incubation with bacteria, bioaerosol filtration efficiency	Bacterial	Untreated	Biobentonite carrier: no antimicrobial activity. Bioperlite carrier: inoculation and bioaerosol tests 95% of *E. coli*, 65.5% of *S. aureus* ‘blocked’
Ren et al. (2018) [32]	NIOSH N95 FFR	N-halamine: MC	Incubation with bacteria, bioaerosol challenge	Viral	Ethanol-soaked fabric	Virus undetectable after 30 min contact. As effective as sodium hypochlorite
Demir et al. (2015) [22]	PP nonwoven	N-halamine: MC	Incubation with bacteria, bioaerosol challenge	Bacterial	Untreated	No viable bacteria recovered from treated fabrics or pores
Duong-Quy (2020) [29]	Prototype	Plectranthii amboinicii plant oil extract	Subject mask wearing: Bacterial inhibition, aerobic microbial test	Recovered bacteria	Conventional surgical mask	Both conventional and LMC showed sterile rings indicating both resistant to bacteria, no significant difference in radius. Antibacterial ability greater for aerobic microbial testing
Woo et al. (2012) [31]	Filters	DAS	Bioaerosol challenge	Viral	Untreated	Very low survivability of MS2 on all filter types treated with DAS. Higher concentration of DAS associated with lower survivability

FFP = Filtering Face Piece. FFR = Filtering Facepiece Respirator. NIOSH = US National Institute for Occupational Safety and Health. RH = room humidity. MC = 1-Chloro-2,2,5,5-tetramethyl-4-imidazolidinone (a N-halamine monochlorinated compound). LIG = laser induced graphene. DAS = dialdehyde starch. PP = polypropylene. CF = cellulose filter. PF = polypropylene filter. QAC = quaternary ammonium compound. SEM = scanning electron microscopy. TEM = transmission electron microscopy. FESEM = field emission scanning electron microscopy.

**Table 2:** Secondary outcomes of included studies.

	Safety	Breathability	Filtration Efficiency	Stability/Durability	Reusability	Cost/Production
Borkow et al. (2010) [17]	Copper eluted to air from test mask in 5 h: 0.467 ± 0.47 pg (<10^5^ folds lower than permissible exposure limit)		Filtration efficiency unaffected by treatment			Statement: copper oxide layer does not add ‘significant costs’
Li et al. (2006) [18]	No sign of skin allergy/irritation after 1 h 15 min wearing (20 volunteers)					
Zheng et al. (2016) [24]						
Hiragond et al. (2018) [20]						Statement: starch is abundant and low cost
Rengasamy et al. (2010) [16]				Antiviral activity only observed at high temp and RH		
Kumar et al. (2021) [26]		Pressure drop similar for treated mask at low velocity, slight increase at high velocity	Filtration efficiency unaffected by treatment		Rejection efficiency unchanged after multiple treatment cycles. Nonwetting surface properties grant ‘self-cleaning’ ability	Statement: reliable and suitable for industrial production
Lore et al. (2012) [25]		Elevated pressure drop	Filtration efficiency unaffected by treatment			
Rubino et al. (2020) [27]	Statement: Salt types safe	No significant rise in pressure drop	Filtration efficiency improved by treatment	Environmental stability: stored at 37 °C, 70, 80, 90% RH for 5 days, antimicrobial properties improved	Statement: ‘safe reusability without further processing’	Statement: salt types are inexpensive; production cost would be lower compared with melt blowing methods
Quan et al. (2017) [30]				Environmental stability: 37 °C 70% RH storage did not affect efficacy	Statement: reusable at normal environmental conditions	Statement: treatment is low-cost
Huang et al. (2020) [28]		Pressure drop similar for proposed system and MBF standard			‘Self-reporting of mask conditions’: through response to moisture	Statement: LIG can be created using wide range of carbon precursors allowing easy supply
Tseng et al. (2006) [19]			Filtration efficiency unaffected by treatment	‘Decontamination test’ challenging masks with pathogens repeated 1, 2, 4 or 8 days after coating-Efficacy maintained		
Xiong et al. (2021) [21]		Air permeability decreased with increasing nanocomposite loading. Acceptable at 10% (114.9 mm/s). PM2.5 removal efficiency >90%			Thermal conductivity as proxy for reusability: maintained after 5 cycles of reuse, PM2.5 removal efficiency also unaffected	
Majchrzycka et al. (2012) [23]			Filtration efficiency unaffected by treatment			Industrial synthesis: Found to be as effective as laboratory
Ren et al. (2018) [32]	Statement: MC has low toxicity					Statement: ‘coating procedure is straightforward and inexpensive’
Demir et al. (2015) [22]	Statement: ‘no issues of biocompatibility or toxicity’ (MC is not volatile and does not emit chlorine gas)	Air permeability not affected by treatment		Antimicrobial system deactivated by fluorescent light—storage implications		
Duong-Quy (2020) [29]	Nitric oxide as a biomarker for respiratory inflammation induced by mask–lower in subjects wearing test mask than conventional mask	Subjects reported higher breathability of test mask than conventional mask				Using natural compound; renewable manufacturing
Woo et al. (2012) [31]	Statement: DAS does not release toxic chemicals	Pressure drop: air resistance reduced in CFs but not PF				

RH = room humidity. MC = 1-Chloro-2,2,5,5-tetramethyl-4-imidazolidinone (a N-halamine monochlorinated compound). LIG = laser induced graphene. DAS = dialdehyde starch. CF = cellulose filter. PF = polypropylene filter.

## Supplementary Materials

The following are available online at https://www.mdpi.com/article/10.3390/jcm10184066/s1, Table S1. A list of excluded studies and reasons for their exclusion, Table S2: Detailed information extracted from included studies, Table S3. Risk of bias tool, defining low, moderate and high risk of bias for each category, Table S4. Results of risk of bias assessment for each area and overall risk of bias score.
