# Supplementary material for: Enhanced Medical and Community Face Masks with Antimicrobial Properties: A Systematic Review
Source: J Clin Med. 2021 Sep 9;10(18):4066. doi: 10.3390/jcm10184066 (PMC8472488; doi:10.3390/jcm10184066)
Supplement: Supplementary file 1 [file jcm-10-04066-s001.zip › jcm-1349690-supplementary.pdf]

Evaluated string: (((((((((respirator) OR (mask)) OR (filtering)) OR (nonwoven)) OR (fabric)) OR (electro AND spun)) OR (textile)) OR (personal AND protection AND equipment)) AND ((((((((antimicrobial) OR (antiviral)) OR (nanoparticles)) OR (nanotechnology)) OR (viricidal)) OR (biocidal)) OR (bactericidal))

S1: Search string used to retrieve hits from databases

	Title	Journal	Year	DOI	Reason for exclusion
Si et al.	Daylight-driven rechargeable antibacterial and antiviral nanofibrous membranes for bioprotective applications	Science Advances	2018	10.1126/sciadv.aar5931	Excluded: Not intended for masks and/or respirators and/or filtering facepiece
Monmaturapoj et al.	Antiviral activity of multifunctional composite based on TiO(2)-modified hydroxyapatite	Mater Sci Eng C Mater Biol Appl	2018	10.1016/j.msec.2018.06.045	Excluded: Not intended for masks and/or respirators and/or filtering facepiece
Catel-Ferreira et al.	Antiviral effects of polyphenols: development of bio-based cleaning wipes and filters	J Virol Methods	2015	10.1016/j.jviromet.2014.10.008	Excluded: Not intended for masks and/or respirators and/or filtering facepiece
Tang et al.	Daylight-Induced Antibacterial and Antiviral Cotton Cloth for Offensive Personal Protection	ACS Appl Mater Interfaces	2020	10.1021/acsami.0c15540	Excluded: Not intended for masks and/or respirators and/or filtering facepiece
Zhanget al.	Daylight-Induced Antibacterial and Antiviral Nanofibrous Membranes Containing Vitamin K Derivatives for Personal Protective Equipment	ACS Appl Mater Interfaces	2020	10.1021/acsami.0c14883	Excluded: Not intended for masks and/or respirators and/or filtering facepiece
Joe et al.	Evaluation of Ag nanoparticle coated air filter against aerosolized virus: Anti-viral efficiency with dust loading	J Hazard Mater	2016	10.1016/j.jhazmat.2015.09.017	Excluded: Not intended for masks and/or respirators and/or filtering facepiece
Salussoglia et al.	Evaluation of filter media covered with spun fibres and containing thyme essential oil with antimicrobial properties	Environ Technol	2020	10.1080/09593330.2020.1786167	Excluded: Not intended for masks and/or respirators and/or filtering facepiece
Khanzada et al.	Fabrication of Promising Antimicrobial Aloe Vera/PVA Electrospun Nanofibers for Protective Clothing	Materials (Basel)	2020	10.3390/ma13173884	Excluded: Not intended for masks and/or respirators and/or filtering facepiece
Choi et al.	Herbal Extract Incorporated Nanofiber Fabricated by an Electrospinning Technique and its Application to Antimicrobial Air Filtration	ACS Appl Mater Interfaces	2015	10.1021/acsami.5b07441	Excluded: Not intended for masks and/or respirators and/or filtering facepiece
Monge et al.	Highly Effective Inactivation of SARS-CoV-2 by Conjugated Polymers and Oligomers	ACS Appl Mater Interfaces	2020	10.1021/acsami.0c17445	Excluded: Not intended for masks and/or respirators and/or filtering facepiece
Umar et al.	Highly potent silver-organoalkoxysilane antimicrobial porous nanomembrane	Nanoscale Res Lett	2013	10.1186/1556-276X-8-164	Excluded: Not intended for masks and/or respirators and/or filtering facepiece
Tatzber et al.	Improved protection of filtering facepiece through inactivation of pathogens by hypertonic salt solutions - A possible COVID-19 prevention device	Prev Med Rep	2020	10.1016/j.pmedr.2020.101270	Excluded: Not intended for masks and/or respirators and/or filtering facepiece
Kozlova et al.	Inactivation and mineralization of aerosol deposited model pathogenic microorganisms over TiO2 and Pt/TiO2	Environ Sci Technol	2010	10.1021/es100156p	Excluded: Not intended for masks and/or respirators and/or filtering facepiece
Tatzber et al.	Improved protection of filtering facepiece through inactivation of pathogens by hypertonic salt solutions – A possible COVID-19 prevention device	Preventive Medicine Reports	2020	10.1016/j.pmedr.2020.101270	Excluded: Not intended for masks and/or respirators and/or filtering facepiece

Fujimori et al.	Novel antiviral characteristics of nanosized copper(I) iodide particles showing inactivation activity against 2009 pandemic H1N1 influenza virus	Appl Environ Microbiol	2012	10.1128/AEM.06284-11	Excluded: Not intended for masks and/or respirators and/or filtering facepiece
Chen et al.	Antiviral Activity of Graphene-Silver Nanocomposites against Non-Enveloped and Enveloped Viruses	Int J Environ Res Public Health	2016	10.3390/ijerph13040430	Excluded: Not intended for masks and/or respirators and/or filtering facepiece
Ali et al.	Performance of silver, zinc, and iron nanoparticles-doped cotton filters against airborne E. coli to minimize bioaerosol exposure	Air Qual Atmos Health	2018	10.1007/s11869-018-0622-0	Excluded: Not intended for masks and/or respirators and/or filtering facepiece
Wang et al.	Preparation and characterization of the anti-virus and anti-bacteria composite air filter materials	Sci China Technol Sci	2013	10.1007/s11431-012-5069-1	Excluded: Not intended for masks and/or respirators and/or filtering facepiece
Jung et al.	Preparation of airborne Ag/CNT hybrid nanoparticles using an aerosol process and their application to antimicrobial air filtration	Langmuir	2011	10.1021/la201851r	Excluded: Not intended for masks and/or respirators and/or filtering facepiece
Abdurazova et al.	Preparation of photochemical coatings of metal films (copper, silver and gold) on dielectric surfaces and studying their antimicrobial properties	Colloids and Surfaces A	2017	10.1016/j.colsurfa.2017.06.070	Excluded: Not intended for masks and/or respirators and/or filtering facepiece
Versoza et al.	Solid oxygen-purifying (SOP) filters: A self-disinfecting filters to inactivate aerosolized viruses	Int. J. Environ. Res. Public Health	2020	10.3390/ijerph17217858	Excluded: Not intended for masks and/or respirators and/or filtering facepiece
Gopal et al.	Zinc-embedded fabrics inactivate SARS-CoV-2 and influenza A virus	bioRxiv	2020	10.1101/2020.11.02.365833	Excluded: Not intended for masks and/or respirators and/or filtering facepiece
Wong et al.	In Vivo Protective Performance of N95 Respirator and Surgical Facemask	Am J Ind Med.	2006	10.1002/ajim.20395	Excluded: No test made on the antimicrobial efficacy
Imani et. Al	Antimicrobial Nanomaterials and Coatings: Current Mechanisms and Future Perspectives to Control the Spread of Viruses Including SARS-CoV-2	ACS Nano	2020	10.1021/acsnano.0c05937	Excluded: No test made on the antimicrobial efficacy
Almeida et al.	Antimicrobial Photodynamic Therapy in the Control of COVID-19	Antibiotics	2020	10.3390/antibiotics9060320	Excluded: No test made on the antimicrobial efficacy
Kumar Raghav et al.	Are graphene and graphene-derived products capable of preventing COVID-19 infection?	Med Hypotheses	2020	10.1016/j.mehy.2020.110031	Excluded: No test made on the antimicrobial efficacy
Otto et al.	Layer-by-Layer Nanocoating of Antiviral Polysaccharides on Surfaces to Prevent Coronavirus Infections	Molecules	2020	10.3390/molecules25153415	Excluded: No test made on the antimicrobial efficacy
Palmieriet al.	Can graphene take part in the fight against COVID-19?	Nano Today	2020	10.1016/j.nantod.2020.100883	Excluded: No test made on the antimicrobial efficacy
Chauhanet al.	Comprehensive Review on Current Interventions, Diagnostics, and Nanotechnology Perspectives against SARS-CoV-2	Bioconjug Chem	2020	10.1021/acs.bioconjchem.0c00323	Excluded: No test made on the antimicrobial efficacy
Sachan et al.	COVID-19 Pandemic Has Spurred Materials Researchers to Develop Antiviral Masks	ACS Cent Sci	2020	10.1021/acscentsci.0c01172	Excluded: No test made on the antimicrobial efficacy
O'Dowd et al.	Face Masks and Respirators in the Fight against the COVID-19 Pandemic: A Review of Current Materials, Advances and Future Perspectives	Materials	2020	10.3390/ma13153363	Excluded: No test made on the antimicrobial efficacy
Chua et al.	Face Masks in the New COVID-19 Normal: Materials, Testing, and Perspectives	Research	2020	10.34133/2020/7286735	Excluded: No test made on the antimicrobial efficacy
Campos et al.	How can nanotechnology help to combat COVID-19? Opportunities and urgent need	J Nanobiotechnology	2020	10.1186/s12951-020-00685-4	Excluded: No test made on the antimicrobial efficacy

De Sio et al.	Personalized Reusable Face Masks with Smart Nano-Assisted Destruction of Pathogens for COVID-19: a Visionary Road	Chemistry	2020	10.1002/chem.202004875	Excluded: No test made on the antimicrobial efficacy
Toledo et al.	Promising Nanostructured Materials against Enveloped Virus	An Acad Bras Cienc	2020	10.1590/0001-3765202020200718	Excluded: No test made on the antimicrobial efficacy
Chowdhury et al.	Prospect of biobased antiviral face mask to limit the coronavirus outbreak	Environ Res	2021	10.1016/j.envres.2020.110294	Excluded: No test made on the antimicrobial efficacy
Ahmed et al.	Protecting healthcare workers during COVID-19 pandemic with nanotechnology: A protocol for a new device from Egypt	J Infect Public Health	2020	10.1016/j.jiph.2020.07.015	Excluded: No test made on the antimicrobial efficacy
Pemmada et al.	Science-Based Strategies of Antiviral Coatings with Viricidal Properties for the COVID-19 Like Pandemics	Materials	2020	10.3390/ma13184041	Excluded: No test made on the antimicrobial efficacy

Table S1. A list of excluded studies and reasons for their exclusion

	Substrate	Type of antimicrobial system	Method of integration	Antimicrobial efficacy testing methods	Pathogen(s) used in testing	Comparators/ controls	Antimicrobial efficacy results
Borkow et al (2010)[17]	NIOSH N95 FFP	Copper oxide (CuO)	Impregnation of fabric layers (three out of four mask layers) with CuO particles	In vitro testing: Bioaerosol challenge modified ASTM Method F 2101.01, Bacterial Filtration Efficacy (BFE)	Viral: Human influenza A virus (A/Puerto Rico/8/34 (H1N1)), Avian influenza virus (Turkey/Wis/66 (H9N2))	Similar FFP N95 without CuO impregnation	Viral filtration: No statistical difference between control and test mask. Virus deactivation: Statistically significant higher direct contact inactivation in test masks than control 2.88 log for H1N1, 3.13 log for the H9N2
Li et al (2006)[18]	FFP	Silver nitrate and titanium dioxide nanoparticles	Coating with nanoparticle emulsion onto one side of the outermost hydrophobic mask layer	Inoculation of fabric with pathogens: AATCC 100-1999. AATCC Test Method 100-1999, assessment of antibacterial finishes on textile materials	Bacterial: *Escherichia coli, Staphylococcus aureus*	Sterile FFP with no bacterial inoculation	Control mask: Increase in viable counts of *E. coli* and *S. aureus*, 25% and 50%<br>Test mask: 100% reduction in viable *E. coli* and *S. aureus*
Zheng et al (2016)[24]	Nonwoven fabric samples from middle layer of particulate respirator 3M N95 (NIOSH approved)	Silver nanoparticles	Initial surface coating with sodium oleate, subsequent coating with silver nitrate solution	Bacterial growth track following inoculation, FESEM examination of fabrics post inoculation	Bacterial: *Pseudomonas aeruginosa, Staphylococcus aureus*	Uncoated fabric	Growth was effectively inhibited for both pathogen challengers. By FESEM: Few bacterial cells intact on coated surface, many on control. Debris on coated surface indicates cellular disruption of pathogens
Hiragond et al (2018)[20]	Commercially available surgical face mask (non-woven)	Colloidal silver nanoparticles	Masks were soaked in solutions of known concentration (50 ppm and 100 ppm) for 5-7 hours and dried for 4-5 hours at room temperature	Well diffusion assay – measure of inhibition zone	Bacterial: *Escherichia coli, Staphylococcus aureus*	Untreated mask	Inhibition zone of treated masks significantly higher than control.<br>Increased inhibition for 100ppm sample.
Rengasamy et al (2010)[16]	Circular cut-outs from 4 antimicrobial FFRs from manufacturers: Not approved by US regulatory agencies for antimicrobial efficacy	Silver-copper, EvixO_3-Shield technology, Iodinated resin, Titanium dioxide	Embedded into filter fibre of outer layer. Outer layer incorporation. Filtering layer incorporation. Filtering layer coating	Bioaerosol challenge using Respirator Test System to circular mask cut-outs. Conditions: 1) 22°C 30%RH for 0, 8, 20h, 2) 37°C 80%RH for 0, 2, 4h	Viral: MS2	NIOSH-approved P95 particulate respirator (similar cut-out)	Conditions: 1) 1-$\log_{10}$ reduction up to 20 hours, no significant difference to control 2) Silver-copper and EvixO_3-Shield technology higher $\log_{10}$ reduction than control. Highest: EvixO_3-Shield technology, 3.7-$\log_{10}$ reduction in 4 hours
Kumar et al (2021)[26]	PP nonwoven fabrics used to make commercial surgical masks	Photoactive copper nanoparticles (Cu NPs) enhanced with Shellac adhesive	Coated using specifically developed: 3D microfluidic spray device	Bacterial inoculation and five-minute incubation followed by fluorescence microscopy to determine viable growth. Bacterial live dead inoculation assay using green/red fluorescent strain. Bioaerosol of VLPs: viable particles found up/downstream of sample were	Bacteria: *Escherichia coli (*Modified to allow fluorescence (1 emits green fluorescence, 1 green/red for alive/dead)). Viral: Virus like particles similar in size and content to COVID-19 (Extracellular vesicles)	Untreated fabric	4-log reduction in *E. coli* CFUs. Fluorescence assays of bacterial viability and live/dead indicate >99.99% reduction of *E. coli*. VLP concentration on the treated fabric decreased by 2-3 log, however negligible reduction was found on untreated fabric samples Photoactivity proved through observed degradation

				counted using nanoparticle tracking. Photoactivity evaluated through degradation of methyl blue			of methyl blue on addition to treated fabric
Lore et al (2012)[25]	Samples of four commercially available NIOSH-approved FFRs	Iodine-based biocide	N/A – commercially available masks used	Bioaerosol challenges – measurement of viable concentration (based on log_{10} inactivation) of pathogen upstream and downstream of each test sample	Bacteria: *Bacillus atrophaeus* vegetative bacteria and endospores. Viral: MS2	FFRs with equivalent inherent physical filtration efficiency. Also controlled for presence of iodine vapor capture using an iodine neutralizer in impinger medium	No detectable antimicrobial properties in test masks compared with conventional
Rubino et al (2020)[27]	PP fibres of large-pore membranes. Target: three-ply surgical masks (Fisherbrand Facemasks; Fisher Scientific, Pittsburgh, PA, USA)	Salts: sodium Cloride (NaCl), potassium sulfate ($K2SO_4$) potassium chloride (KCl)	Salt solutions applied to inner layer of fabric and left to dry	Bioaerosol challenge: tested the time-dependent inactivation of pathogens incubated on coated surface and confirmed by TEM. In vivo: Bacteria were recovered from salted fabrics and infected into mice	Bacteria: *Klebsiella pneumoniae,* methicillin-resistant, *Staphylococcus aureus*, *Escherichia coli, Pseudomonas aeruginosa, Streptococcus pyogenes*	Untreated fabric	Physical damage caused to all pathogen challengers. Time-dependent bacterial inactivation seen for all salt types. Mice infected with bacteria from treated fabric lost less body weight and demonstrated lower concentrations of lung bacteria than those infected with pathogen from untreated fabric
Quan et al (2017)[30]	Filtration layer within three-ply surgical mask, polypropylene (PP) microfiber filter	Salt: Sodium chloride (NaCl)	Coated using NaCl solution containing surfactant to enhance coating of hydrophobic PP fibres	Bioaerosol filtration efficiency: virus penetration measured using TEM	Viral: H1N1 pandemic influenza virus (A/California/04/2009, abbreviated as CA/09), A/Puerto Rico/08/1934 (PR/34 H1N1), A/Vietnam/ 1203/2004 (VN/04 H5N1)	Untreated standard surgical mask filter	All challenge viruses were inactivated. Evidence that this is due to hyperosmotic stress on viral envelope
Huang et al (2020)[28]	LIG: Hydrophobic (150° contact angle) + Hydrophilic (20° contact angle)	Inherent from LIG	10.6µm CO_2 laser converts 0.05 mm thick Polyimide substrate into porous graphene	Bacterial inhibition rate: inoculation of samples with *E. coli*, live/dead assay, SEM imaging. Bioaerosol collection: bacteria collected from air using LIG or MBF as a filter	Bacteria: *Escherichia coli* Fluorescent strain for live/dead, Staphylococcus epidermidis	Filter layer of commercial activated carbon face mask and surgical masks: Activated carbon fibre (ACF) and melt-blown fabrics (MBF)	Antibacterial activity against *E. coli*: LIG: 81.57%, ACF: 2.00%, MBF: 9.13%. SEM shows surface disruption of *E. coli* recovered from LIG. Staphylococcus epidermidis showed how bacterial particulates accumulate on mask. Aerosolized bacterial efficiency of LIG was estimated as 88.89%. *E. coli* viability dropped from 1.9×10^6 to 0.35×10^6 CFU/mL after 8 h (corresponding to 0.73 log reduction). Similar activity for hydrophilic and hydrophobic versions
Tseng et al (2006)[19]	Surgical mask (AERO PRO, Shuenn Bao Shing Co., Chang Hua, Taiwan)	Goldshield 5 (GS5; quaternary ammonium agent, AP Goldshield LLC, USA)	Spray coating of GS5 suspension individually onto 3 filtering mask layers	Bioaerosol testing: aerosol deposition onto surface	Bacterial: *Acinetobacter baumannii, Enterococcus faecalis, Staphylococcus aureus*	Untreated mask	>99.3% antimicrobial efficiency against bacteria on mask surface for all test pathogens

Xiong et al (2021)[21]	Prototype	Functionalised 3-(Trimethoxysilyl)-propyl-dimethyloctadecyl ammonium chloride solution QAC with hexagonal boron nitride (h-BN) nanoparticles (Hexagonal boron nitride (h-BN) nanoparticles	QAC were immobilised onto an activated surface of PP ultrafine fibre nonwovens, forming covalent bonds with h-BN nanoparticles. This surface was further soaked with QAC/h-BN platelet suspension 3, 5, 7, and 10 wt % nanocomposite concentration	Incubation of test sample with pathogen challengers (ISO 22196 and JIS Z 2801), Zone of inhibition	Bacterial: *Escherichia coli* (Carolina #155065A), *Staphylococcus aureus* (Carolina #155556)	Untreated fabric	Antibacterial rate 99.3% for *E. coli* and 96.1% for *S. aureus* through 'contact killing' mechanism
Majchrzycka et al (2012)[23]	Fabric: polypropylene melt-blown filtering nonwovens	Alkylammonium microbiocide in two nonorganic carriers: perlite (Bioperlite) and bentonite (Biobentonite)	During fibre synthesis: biocidal agents introduced in powder to mass of developed polypropylene fibres through fibre-forming head	Inoculation and incubation with pathogen challengers. Bioaerosol filtration efficiency: samples sprayed, bacteria removed and counted from sample and from microbiological filter behind sample	Bacterial: *Escherichia coli* (ATCC 8739), *Staphylococcus aureus* (ATCC 6538)	Nonwoven fabrics with Perlite or Bentonite that were not charged with any biocide	Biobentonite did not display antimicrobial activity – no significant difference in bacterial survivability. Bioperlite did pertain antimicrobial activity through inoculation and bioaerosol tests (95% of *E. coli*, 65.5% of *S. aureus* 'blocked' by sample)
Ren et al (2018)[32]	Fabric samples (nonwoven) from NIOSH certified N95 respirator (3 M, 1860)	N-halamine: MC	Soaking of coupon samples in solution of MC at known concentration, air dried	'Sandwich test' incubate samples with pathogen challengers. Bioaerosol challenge: Recover virus from samples after subject to aerosol	Viral: AI H1N1 virus	Sample fabric coupon soaked in ethanol only. Sodium hypochlorite (disinfectant)	MC significantly inactivated virus relative to control. Virus undetectable after 30 minutes contact. As effective as sodium hypochlorite
Demir et al (2015)[22]	Fabrics: surgical and N95 types of polypropylene nonwovens (commercially available)	N-halamine: MC	Fabric coating with MC EtOH solution to leave adsorbed layer	'Sandwich test' incubate samples with pathogen challengers. Bioaerosol challenge: bacteria collected in pores behind fabric sample	Bacterial: *Escherichia coli, Staphylococcus aureus*	Untreated fabric	Significant bacterial reduction relative to control. No viable bacteria recovered from treated fabrics or pores (aerosol challenge). Differences between pathogens due to morphology determined penetration
Duong-Quy (2020)[29]	Lamdong Medical College (LMC) mask: 7 layers. External layer: activated carbon, standardized BSI and Plectranthii amboinicii plant oil extract 0.5%	Plectranthii amboinicii plant oil extract	Treated layers placed in solution of BSI and Plectranthii amboinicii plant oil extract	Bacterial inhibition: bacteria recovered from each face mask before and after use was grown. Aerobic microbial test: disc implants of masks were compared for bacterial growth	Not tested on specific pathogen, bacteria were recovered before and after wearing of masks by subjects	Conventional surgical mask (GreetMed, ANJ International Corp., UK).	Both conventional and LMC showed sterile rings indicating both resistant to bacteria, no significant difference in radius. Antibacterial ability greater in for aerobic microbial testing
Woo et al (2012)[31]	Two CFs that are commonly used for air cleaning and a PF	DAS	Filters were modified by application with a DAS aqueous suspension	Bioaerosol: MS2 nebulised in artificial saliva applied to filters	Viral: MS2	Untreated filters	Very low survivability of MS2 on all filter types treated with DAS. Higher concentration of DAS associated with lower survivability

Table S2: Detailed information extracted from included studies

FFP = Filtering Face Piece. FFR = Filtering Facepiece Respirator. NIOSH = US National Institute for Occupational Safety and Health. RH = room humidity. MC = 1-Chloro-2,2,5,5-tetramethyl-4-imidazolidinone (a N-halamine monochlorinated compound). LIG = laser induced graphene. DAS = dialdehyde starch. PP = polypropylene. CF = cellulose filter. PF = polypropylene filter. MS2 = MS2 bacteriophage virus. BSI = Benzoic acid, Salicylic and lode. QAC = quaternary ammonium compound. SEM = scanning electron microscopy. TEM = transmission electron microscopy. FESEM = field emission scanning electron microscopy.

	Outcome	Low Risk (0 points)	Moderate Risk (1 point)	High Risk (2 points)	Unclear*
**Design**	All	Controlled study or pre-post design	Partially controlled, quasi-experimental, or case-control design	Uncontrolled and no pre-post design	Insufficient information
**Methodological Consistency**	Germicidal	Detailed description of viral/bacterial inoculation procedure (including inoculation medium and transmission mode) AND assay procedure (including times/temperatures where applicable); procedures consistent across arms unless they are experimental variables	Some of the stated criteria not reported	None of the stated criteria reported	Insufficient information
**Population Heterogeneity**	All	Masks or mask components derived from the same batch/lot AND each model analyzed separately	Different mask models analyzed separately but lots not specified	None of the stated criteria reported	Insufficient information
**Sampling Bias**	All	No indication that any masks were excluded from tests OR clear indication of exclusion with analyses or conclusions accounting for the missing information (e.g. sensitivity analysis)	Data is missing for some samples and is not accounted for in analysis or conclusions	Some samples are replaced by new masks (e.g. if untestable due to degradation) and the exclusion of the original samples is not accounted for in analysis or conclusions	Insufficient information
**Selective Reporting**	All	Results for all specified objectives and tests are reported and/or missing data is clearly accounted for in analysis; all results that are reported are mentioned in the methods/objectives	Results of all objectives/tests are included or accounted for but results that were not specified in the methods/objectives are also reported	Results for some objectives/tests are missing and not accounted for	Insufficient information
**Overall Study Risk of Bias (Sum Score)**		**Low Risk of Bias (sum score 0-1)**	**Moderate Risk of Bias (sum score 2-6)**	**High Risk of Bias (sum score 7-12)**	

Table S3. Risk of bias tool, defining low, moderate and high risk of bias for each category

	Design*	Methodology*	Population Heterogeneity*	Sampling Bias*	Selective Reporting*	Overall ROB (Score)*	Peer-Reviewed†
**Germicidal**							
Borkow et al (2010)[17]	Low	Low	Moderate (no mention of batch/lot)	Low	Low	**Low (1)**	Yes
Li et al (2006)[18]	Low	Low	Moderate (no mention of batch/lot)	Low	Low	**Low (1)**	Yes
Tseng et al (2006)[19]	Low	Low	Moderate (no mention of batch/lot)	Low	Low	**Low (1)**	Yes
Hiragond et al (2018)[20]	Low	Moderate (no bacterial strain info)	High (no detail of mask)	Low	Low	**Moderate (3)**	Yes
Rengasamy et al (2010)[16]	Low	Low	Moderate (no mention of batch/lot)	Low	Low	**Low (1)**	Yes
Xiong et al (2021)[21]	Low	Low	Low (synthesise their own fabric)	Low	Low	**Low (0)**	Yes
Demir et al (2015)[22]	Low	Low	Moderate (no mention of batch/lot)	Low	Low	**Low (1)**	Yes
Majchrzycka et al (2012)[23]	Low	Low	Low (synthesise their own fabric)	Low	Low	**Low (0)**	Yes
Zheng et al (2016)[24]	Low	Low	Moderate (no mention of batch/lot)	Low	Low	**Low (1)**	Yes
Lore et al (2012)[25]	Low	Low	Moderate (no mention of batch/lot)	Low	Low	**Low (1)**	Yes
Kumar et al (2021)[26]	Low	Low	Moderate (no mention of batch/lot)	Low	Low	**Low (1)**	Yes
Rubino et al (2020)[27]	Low	Moderate (no strain info, not detailed)	High (no detail of substrate)	Low	Low	**Moderate (3)**	Yes
Huang et al (2020)[28]	Low	Low	Low (synthesise their own fabric)	Low	Low	**Low (0)**	Yes
Duong-Quy (2020)[29]	Low	High (generally unclear)	Low (synthesise their own mask)	Low	Moderate	**Moderate (3)**	Yes
Quan et al (2017)[30]	Low	Low	High (no detail of mask source)	Low	Low	**Moderate (2)**	Yes

	Design*	Methodology*	Population Heterogeneity*	Sampling Bias*	Selective Reporting*	Overall ROB (Score)*	Peer-Reviewed[†]
Woo et al (2012)[31]	Low	Low	Moderate (no mention of batch/lot)	Low	Low	**Low (1)**	Yes
Ren et al (2018)[32]	Low	Low	Moderate (no mention of batch/lot)	Low	Low	**Low (1)**	Yes

Table S4. Results of risk of bias assessment for each area and overall risk of bias score
